# 5-Fluorouracil Toxicity: Revisiting the Relevance of Pharmacokinetic Parameters

**DOI:** 10.3390/ph18050653

**Published:** 2025-04-29

**Authors:** Hans Mielke, Engi Abd Elhady Algharably, Ursula Gundert-Remy

**Affiliations:** 1 Department of Exposure, German Federal Institute for Risk Assessment (BfR), Max-Dohrn 8–10, 10589 Berlin, Germany; hans.mielke@bfr.bund.de; 2 Charité-Universitätsmedizin Berlin, Corporate Member of Freie Universität Berlin and Humboldt Universität zu Berlin, Institute of Clinical Pharmacology and Toxicology, Charitéplatz 1, 10117 Berlin, Germany; ursula.gundert-remy@charite.de

**Keywords:** biologically-based modeling, relationship between AUC and concentration to clinical toxicity, threshold concentrations, therapeutic drug monitoring

## Abstract

**Background/Objectives**: 5-fluorouracil (5-FU) is used in the treatment of solid cancer types. Because of its narrow therapeutic window, drug monitoring is recommended. We were confronted to answer a question on the relevance of concentration as opposed to the area under the plasma concentration–time curve (AUC) to predict toxicity when we had to assess the case of a patient who died after an erroneously high infusion rate. **Methods**: We used physiologically-based pharmacokinetic modeling (PBPK) to simulate the concentration–time profile of 5-FU data on doses, dosing schedules and life-threatening toxicity for both the patient in question as well as data from the literature. Furthermore, steady-state 5-FU concentrations were calculated from an additional set of data found in the literature on AUCs and non-life-threatening toxicity. **Results**: The model predictions matched well with experimental data, confirming the suitability of the model. Life-threatening toxicity was related to a concentration above 6 mg/L, whereas non-life-threatening toxicity was low at concentrations less than 3 mg/L but steeply increased between 3 and 4 mg/L. Data analysis supported by a decision algorithm suggests that the 5-FU steady-state plasma concentration is a better toxicity predictor than the AUC. **Conclusions**: We recommend monitoring the concentration one hour after infusion starts when about 50% of the steady state is reached in patients for whom higher doses are clinically considered relevant. Monitoring the concentration one hour after starting the infusion has the advantage that dose correction could be made early before toxicity can be observed.

## 1. Introduction

5-fluorouracil (5-FU) is a pyrimidine analogue that competitively inhibits the enzyme thymidylate synthase (TS). As a result of intracellular thymine deficiency, DNA synthesis is inhibited causing cytotoxicity, which is the therapeutic goal; however, this is also responsible for the prominent side effects of 5-FU [1]. To a lesser extent, the formation of RNA is also inhibited. 5-FU is the backbone for the therapy of various solid tumors, in particular gastrointestinal cancer [2,3]. 5-FU is metabolized to a great extent (85%) by dihydropyrimidine dehydrogenase (DPD) to the inactive metabolite dihydrofluorouracil [4]. In the European population, partial DPD deficiency is observed with a prevalence of between 3% and 8%, leading to a wide range of drug clearance rates. A complete lack of DPD activity is found in the European population with a prevalence of 0.01 and 0.5% [5]. Because 5-FU has a narrow therapeutic window, non-adapted fluorouracil treatment of patients with DPD deficiency may result in severe and even fatal toxicity [6]. Since 2020, the European Medicines Agency (EMA) recommended that patients should be tested for a lack of DPD before starting cancer treatment with 5-FU via injection or infusion to avoid toxicity [7]. Underdosing is another aspect of the narrow therapeutic range, and therefore, therapeutic drug monitoring (TDM) was recommended by several scientific bodies as well as included in the summary of product characteristics [6,8,9,10]. Additionally, recommendations were issued by the International Association of Therapeutic Drug Monitoring and Clinical Toxicology (IATDMCT) [6]. The pharmacokinetic target parameter, which is used to define the therapeutic range, is the area under the plasma concentration–time curve (AUC). Over some years, the recommended upper value of the therapeutic AUC increased from 20 to 24 mg × h/L to, finally, 20 to 29–30 mg × h/L [11,12,13,14]. Currently, the value 20 to 30–40 mg × h/L is used [15]. When studying the kinetic parameter related to toxicity, nearly all authors focused on the AUC [12,16,17,18,19,20,21,22,23] and did not consider the concentration at the steady state (C_ss_). Van Groeningen at al. [19] reported AUC and C values of patients under treatment with 5-FU; however, they only quantitatively analyzed the relationship between the AUC and toxicity. Ma and colleagues [24] evaluated the dosing rate (mg/h) as well as the dose (mg) to predict 5-FU toxicity in a 2-dimensional fashion. They attributed the outcome of life-threatening toxicity to the combination of a high dose rate and a high dose, with cut-offs of the dose rate of 150 mg/h and that of the dose of 2000 mg [24]. A few studies have demonstrated a strong correlation between the 5-FU C_ss_ and risk of toxicity, supporting its role as a key parameter for therapeutic monitoring. Gamelin et al. [12,25] showed that 5-FU dose adjustment based on plasma levels significantly improved safety, with an optimal therapeutic range identified at 2–3 mg/L. They also highlighted that patients with levels above 3 mg/L (AUC > 24 mg × h/L) were more likely to experience toxicity. Specifically, they concluded that a plasma concentration above 3.4 mg/L would lead to adverse reactions, which were non-life threatening in a greater proportion of patients than concentrations. Yoshida et al. [21] confirmed the significant association between the 5-FU C_ss_, AUC over 3 days and total body clearance with toxicity, although no specific threshold was proposed. Trump et al. [21] further demonstrated that the frequency of stomatitis and leukopenia increased with a higher C_ss_, with stomatitis showing a particularly strong exponential correlation. Grem et al. [26] also analyzed data in a limited number of patients and found that much lower concentrations were related to grade 1 and grade 2 non-lethal toxicity. These findings collectively support C_ss_ as a clinically relevant and practical marker for optimizing 5-FU therapy through TDM.

We faced the question of which pharmacokinetic (PK) parameter is most relevant for predicting drug treatment toxicity when evaluating a patient case. The patient died from sepsis caused by agranulocytosis after receiving a therapeutic dose administered at an erroneously high infusion rate. The total dose administered was 3052 mg 5-FU within two hours instead of 4612 mg 5-FU over a 24 h infusion. Using a physiologically-based pharmacokinetic (PBPK) model, we could demonstrate that the high infusion rate in this patient led to an extremely high maximal concentration, although the simulated AUC was comparable to the AUC to be reached under the planned dosing regimen. This case prompted us to re-evaluate the complex relationship between the dosing, dose and 5-FU toxicity.

Our investigation aims to re-evaluate the recommendations concerning the therapeutic AUC target of 20 to 40 mg/L × h regarding 5-FU safety, noting that these recommendations are not applicable for short i.v. bolus injections or for infusions lasting 120 h or longer [6,15]. No recommendations are provided for short infusions, such as when the majority of the dose is administered within two hours, as in the patient case we studied, instead of over a 24 h period. Moreover, we aimed to investigate the relevance and contribution of the concentration as a PK metric to the risk of toxicity.

## 2. Results

### 2.1. Model Evaluation

**Figure 1:**
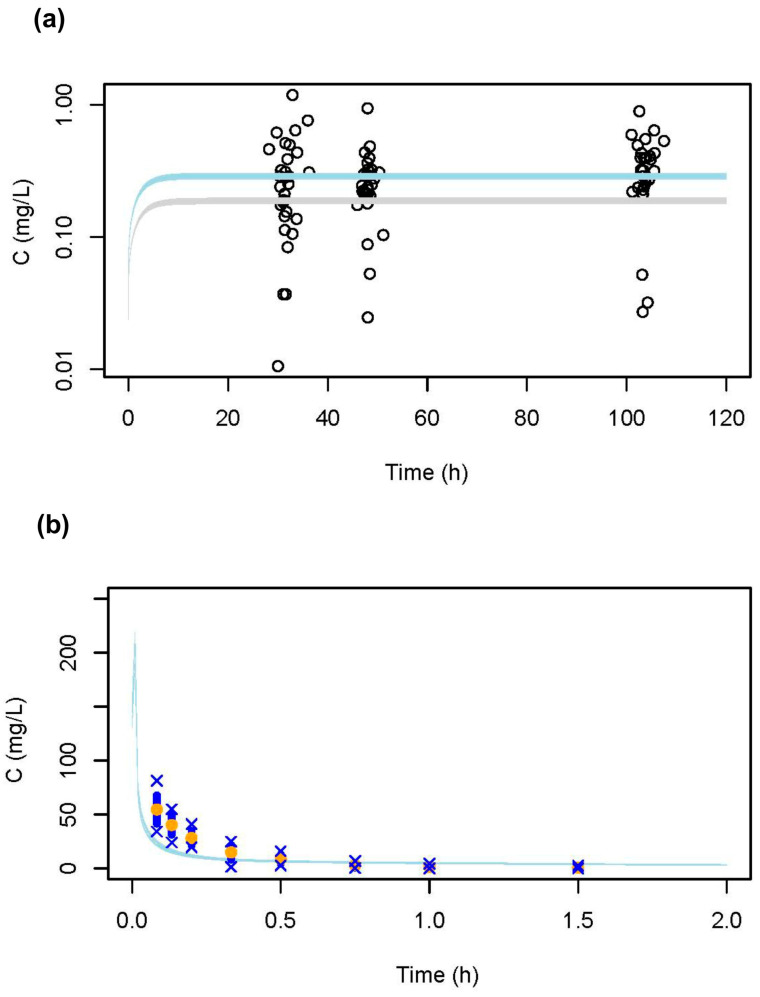
PBPK model evaluation and validation. (**a**) Comparison of the concentration–time data from the population pharmacokinetic study by Arshad et al. [27], with the predicted concentration–time profiles (dose: 650 and 1000 mg/m^2^ as continuous i.v. infusions over 24 h; light blue curve: 650 mg/m^2^; light grey curve: 1000 mg/m^2^). (**b**) Comparison of the predicted concentration–time profile, with the concentration–time data taken from the pharmacokinetic study by Finch et al. [28] after a 1.0 g i.v. bolus dose (yellow and blue dots represent the mean and standard deviation for the observed concentrations, respectively; crosses represent the minimal and maximal observed concentrations). Confidence interval bands reflect inter-subject variability in the clearance range (95% CI: 224–276 L/h) as estimated by [27].

The simulated concentration–time profile was compared with the plasma concentration–time profiles in patients treated with 5-FU. For testing the suitability of our model, we compared model predictions based on doses and the duration of the infusion or the injection, respectively, with the experimental published data. In one study applying a population PK approach, plasma concentrations were displayed in a figure for continuous 5-FU infusion [27]. In this study, two different dosage levels were used, 650 and 1000 mg/m^2^/day as a 24 h continuous i.v. infusion, without indicating in the figure which data points corresponded to the lower or higher dose. Therefore, we simulated the concentration–time profile for both dosage levels. In Figure 1a, the predicted concentration–time profiles are presented together with the experimental data points. The predicted concentration–time profiles match well with the experimental data, thus confirming that the model output provides valid predictions. The model was also able to predict the concentration–time profile following intravenous injections using data from Finch et al. [28] (Figure 1b).

### 2.2. Modeled Css and AUC

#### 2.2.1. Data of the Patient

**Figure 2:**
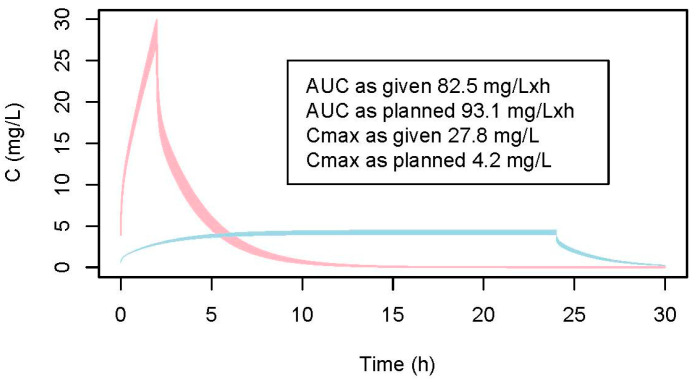
Simulated concentration–time profiles of the patient (49 kg). Blue: planned dosing schedule = 4612 mg 5-FU within 24 h; red: erroneously given = 3052 mg 5-FU within 2 h. Confidence interval bands reflect inter-subject variability in the clearance range (95% CI: 224–276 L/h) as estimated by [27].

In Figure 2, the concentration–time course for the erroneous schedule is depicted as well as the concentration–time course for the planned schedule. The resulting maximal concentration and the AUC for the erroneously administered schedule were 27.7 mg/L and 82.5 mg/L × h, respectively, and for the planned schedule were 4.2 mg/L and 99.5 mg/L × h, respectively. Thus, while the AUCs were nearly comparable, the peak concentration was six-fold higher for the erroneous schedule. Nevertheless, both lie above the upper bound of the therapeutic window.

#### 2.2.2. Data from the Literature

**Figure 3:**
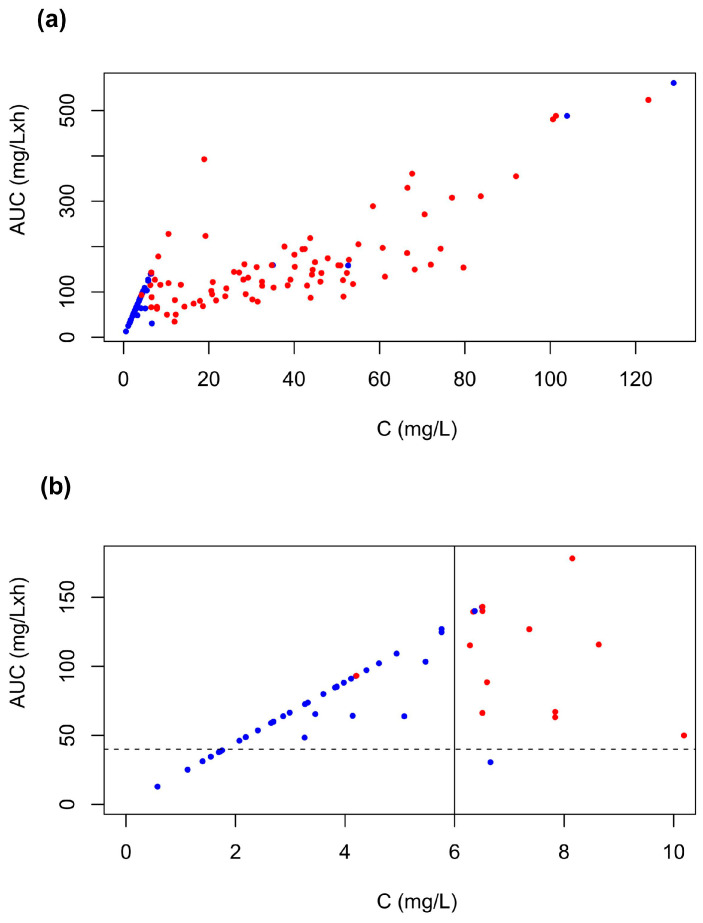
Simulated concentrations (C_ss_) and AUCs with respective toxicity outcomes based on data extracted from Ma et al. [24]. Blue dots: no life-threatening toxicity; red dots: life-threatening toxicity. (**a**) Entire range of simulated C_ss_ values and AUCs; (**b**) enlarged detail from Panel (**a**) expanding simulated concentrations and corresponding AUCs for life-threatening toxicity, highlighting the cut-off for toxicity at C = 6 mg/L (solid line). The dashed line represents the highest level of the AUC currently recommended for dosing schedules.

Using the PBPK model, we simulated a total of 138 C and AUC values for the individuals’ information on the doses, mode and duration of the administration, as provided in Ma et al. [24] In this study, 86 patients had life-threatening toxicity, while 52 patients were reported to show non-life-threatening toxicity symptoms (Figure 3a). Nearly all patients with a modeled C_ss_ below 6 mg/L did not experience life-threatening toxicity, with the exception of one patient, who had a C_ss_ of 4.2 mg/L (Figure 3b). Currently, 40 mg/L × h is the recommended target value; however, in patients without toxicity symptoms, we predicted AUC values as high as 130 mg/L × h, provided that the C_ss_ remained below 6 mg/L. Concentrations exceeding 6 mg/L were related to life-threatening toxicity even when the predicted AUCs were only slightly higher than the recommended target value of 40 mg/L × h. Thus, based on this analysis, the C_ss_ seems to be the more relevant PK parameter to predict life-threatening toxicity than the AUC.

### 2.3. Reported AUCs and Estimated C_ss_

Data from three studies [17,29,30] provided a total of 321 AUC values, for which the corresponding C_ss_ levels were calculated. In all three studies, the dosing regimen was consistent at 1000 mg/m² per 24 h. None of the patients died because of the treatment; however, some patients experienced non-lethal toxicity, such as gastrointestinal toxicity or minor hematotoxicity. In these studies, toxicity was reported in 94 patients, along with their corresponding AUC values. In contrast, no signs of toxicity were observed in 227 patients, each with their respective AUC values. Expectedly, the C_ss_ was linearly related to the AUC given the formula used to derive concentrations from AUC values. The new insight we gained from this analysis is the specific concentrations at which toxicity or the absence thereof was observed in the patients.

In those showing signs of toxicity that was not life threatening, the AUCs varied between 21.8 and 126.3 mg/L × h and the C_ss_ between 0.91 to 5.26 mg/L. For the AUC, the mean ±SD (standard deviation) and median (interquartile range; IQR) were 82.2 ± 21.8 and 77.5 (70.8–90.5) mg/L × h, respectively. For the C_ss_, the mean ± SD and median (IQR) were 3.2 ± 0.91 and 3.23 (2.95–3.97) mg/L, respectively.

In patients without clinical signs of toxicity, the AUCs ranged between 9.5 and 105.8 mg/L × h and the corresponding concentration from 0.39 to 4.41 mg/L. For the AUC, the mean (±SD) and median (IQR) were 58.3 ± 68.1 and 65.1 (44.2–72.7) mg/L × h, respectively. For the C_ss_, the mean (±SD) and median (IQR) were 2.40 ± 2.84 and 2.71 (1.84–3.03) mg/L, respectively.

**Figure 4:**
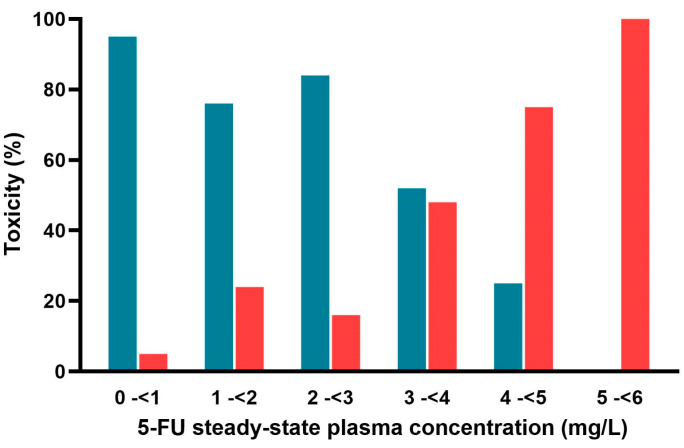
Percentage of patients with or without non-life-threatening effects. Blue: no adverse effect; Red: adverse effect.

We investigated the data to determine the threshold concentration of 5-FU that leads to non-life-threatening toxicity. Hence, we assessed the percentage of occurrences of these toxicity signs in relation to varying concentrations of 5-FU. As can be seen in Figure 4, the small percentage of patients having signs of non-life-threatening toxicity was below a concentration of 3 mg/L. The percentage increased in the concentration range between 3 mg/L and 4 mg/L. The percentage increased further and reached 100% when the concentration ranged between <5 mg/L and 6 mg/L. Concentrations higher than 5.26 mg/L were not found in the publications that served as data sources.

**Figure 5:**
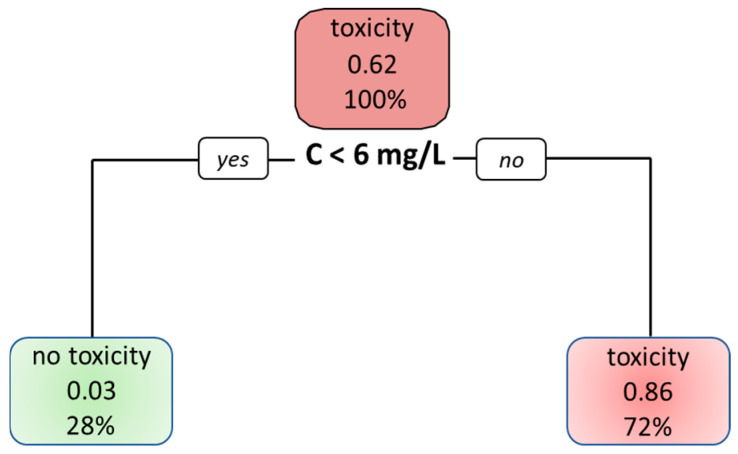
Decision tree, indicating that the concentration is the primary decision endpoint for toxicity over the AUC. The probability of life-threatening toxicity is low (0.03) below a concentration of 6 and high (0.86) above this concentration.

Finally, we constructed a classification tree to utilize the values of the C_ss_ and AUC for the prediction of toxicity. The algorithm selected only the C_ss_ as a relevant parameter and decided that a C_ss_ above 6 mg/L predicts higher probability for life-threatening toxicity and a lower probability below 6 mg/L (Figure 5).

## 3. Discussion

The therapeutic window for 5-FU is narrow, and dosing is complicated by the fact that the metabolism is mediated by the genetically modified activity of DPD. To avoid toxicity, regulatory bodies such as the EMA recommended testing the activity of this enzyme before initiating treatment with 5-FU. Therapeutic drug monitoring is a further step for avoiding over- and underdosing, recommended by several bodies, for example, the IATDMCT. The PK target parameter, which is used to define the therapeutic window, is the AUC over 24 h, which is the usual duration of i.v. administration. It should be noted that the therapeutic window was defined using therapeutic effects as the endpoint, whereas the endpoints of toxicity were weighted less [11,31,32].

The case of a patient who died after an erroneously high infusion rate was the starting point of our investigation. We developed a PBPK model to simulate the concentration–time profile for this patient, using data from the erroneous high infusion rate. We then compared the resulting concentration–time profile and the derived PK parameters, including the peak concentration (C_max_) and AUC, with those that would have been obtained under the planned administration schedule. Compared to other PBPK models, our eight-compartment model focused solely on 5-FU as the parent compound and was developed for a specific clinical context. Similar to previously published 5-FU PBPK models [33,34,35], it supported both IV bolus and infusion routes and incorporates a bone marrow compartment. However, we did not implement tissue-specific DPD, as described by [33], include prodrugs or downstream metabolites, as in [34,35], or describe circadian enzyme rhythms and tumor targeting, as in [34]. Although simpler in structure, only consisting of eight compartments compared to other more complex models [33,34,35], we showed that our model can predict the experimental data with sufficient accuracy (see Figure 1a,b). Hence, the modeled concentration–time profiles can be taken as valid predictions. The data modeled for the patient showed that the AUCs of both administration schedules (3052 mg 5-FU within 2 h, as given, vs. 4612 mg 5-FU within 24 h, as planned) were not much different (82.5 mg/L × h, as given, vs. 99.5 mg/L × h, as planned), the small difference arises from the fact that a smaller dose was administered within two hours, whereas the full dose was planned for a 24 h infusion. Our modeling reflects this difference. However, both AUCs would be above the upper bound of the therapeutic window. In contrast, the highest concentration reached was extremely different between the two regimens, with 27.7 mg/L as given, versus 4.6 mg/L with the planned schedule. The difference in the PK profiles we modeled is supported by experimental data from Fraile et al. [36], who demonstrated very high levels of 5-FU in the plasma and bone marrow following rapid injection but low and sustained levels during a 96-h i.v. infusion.

To investigate whether high concentrations contributed to the unfortunate outcome of the patient in question, we analyzed the literature data on varying 5-FU doses and infusion rates linked to toxicity outcomes. Data from Ma et al. [24] included cases with different dosing schedules and both severe life-threatening and non-life-threatening toxicity, allowing us to simulate concentration–time profiles, calculate AUCs, and correlate these parameters with toxicity. Additionally, data from patients receiving 1000 mg/kg body weight infused over 24 h primarily reporting non-life-threatening toxicity were also evaluated, contrasting with the life-threatening cases in Ma et al. [24]. The latter focused primarily on patient survival or death outcomes. Due to these differences, we evaluated the two datasets separately to examine the relationships between the AUC, concentration and toxicity.

From the analysis of the concentration and AUC derived from the concentration–time profile of Ma et al. [24] related to severe life-threatening toxicity or patients who survived, we could identify a concentration region of 6 mg/L as the threshold for toxicity (Figure 3a,b). Non-life-threatening toxicity occurred in the concentration range below 6 mg/L (with one exception) even if the AUC was as high as 140 mg/L × h. Therefore, we deduced that the C_ss_ would be the better suited parameter for predicting life-threatening toxicity than the AUC. Nevertheless, it is also important to consider the infusion duration, since both the concentration level and its duration are critical determinants of 5-FU toxicity. While prolonged exposure to moderate concentrations may lead to high cumulative exposure (i.e. AUC), short-term exposure to high concentrations can result in acute, potentially life-threatening toxicity.

Data from patients with non-life-threatening toxicity [17,29,30], where all patients received the same treatment schedule with 1000 mg/m^2^ per 24 h, reported AUCs that are statistically different between patients with and without toxicity. When analyzing the percentage of patients with and without non-life-threatening toxic symptoms, we observed a notable trend: within the C_ss_ range of 3.0 to 4.0 mg/L, the percentage of patients with symptoms increased significantly, eventually reaching 100%. This agrees with a phase 2 study involving 18 patients [12] that concluded that plasma concentrations above 3.4 mg/L are associated with a higher incidence of non-life-threatening adverse reactions compared to concentrations below this threshold. Hence, our findings using the larger dataset support this earlier report. On the other hand, Grem et al. [26] reported a rather low concentration (0.07 mg/L) to be associated with grade II toxicity; however, this level was not supported by subsequent reports [17,29,30]. When constructing a partition tree as a decision tool, the resulting tree indicates that the discriminating between the AUC and C_ss_ is a better predictor for life-threatening toxicity, selecting a concentration threshold of 6 mg/L.

Using the AUC as a parameter to guide individual dosing is related to the therapeutic outcome. Typically, when the AUC is measured over 24 h, the concentration would not exceed 1.66 mg/L for a targeted value of 40 mg/L × h (see the Section 4 for the formula). This concentration is significantly lower than those associated with lethal toxicity. On the other hand, in cases in which higher doses are considered therapeutically adequate, the resulting C_ss_ will increase and might exceed the concentrations where adverse effects might occur. Excluding dosing errors, situations involving higher doses or reduced clearance can occur in clinical practice, potentially leading to elevated 5-FU exposure, even though the exact percentage of patients experiencing toxicity under standard therapeutic dosing is not well established. Several factors can contribute to increased systemic exposure to 5-FU and potentially elevated C_ss_ levels. These include DPD deficiency, resulting from specific genetic variants such as DPYD*2A or c.2846A > T, as well as co-medications inhibiting DPD activity or reducing 5-FU metabolism [9]. In these cases, early concentration monitoring during the course of treatment would be a helpful aid for guiding dosing. In this regard, monitoring the concentration of 5-FU could be beneficial in addition to monitoring and assessing the AUC. There is currently no consensus regarding the optimal timing for TDM sampling of 5-FU [22,23]. While some dose adjustment algorithms use early sampling (e.g., 4–8 h after starting infusion), most contemporary protocols recommend sampling at the steady state, typically between 18 and 24 h into a continuous infusion [22,23]. This approach is supported by multiple studies and international guidelines and is common in the current application [37].

We have shown that the risk of non-life-threatening toxicity will increase steeply with a C_ss_ of more than 3 mg/L (Figure 4), and life-threatening toxicity will increase with a C_ss_ of more than 6 mg/L (Figure 3). Monitoring the concentration should be performed one hour after the start of the infusion. Having the terminal half-life of 5-FU estimated from the modeling and confirmed with urinary excretion data by [38], which is longer than the half-life reported, one hour post-infusion start time would be an appropriate time point to measure the plasma concentration. At this time point, about 50% of the C_ss_ would be reached, allowing us to predict the concentration at the steady state (100%). At this early time point, we refer to the dynamic concentration (C_dyn_), as steady-state conditions (C_ss_) have not yet been reached. The aim is to use this concentration to predict the C_ss_, which would finally be reached, making it possible to modify the dosing schedule by reducing the infusion rate to avoid levels above which—as shown by our analysis—toxicity may occur. C_ss_ conditions are not typically achieved until several hours into the infusion. Moreover, based on our analysis, we propose threshold levels that may indicate a risk of toxicity if the infusion were to continue at the same dosing rate. Hence, Cdyn values above 1.5 mg/L at this stage are likely predictive of a subsequent C_ss_ exceeding 3 mg/L, which are associated with non-lethal toxicity, while values above 3 mg/L are predictive of a C_ss_ above 6 mg/L, a level related to lethal toxicity.

Monitoring the concentration at this point in time could provide a basis for the decision whether to continue with the chosen dose or reduce it. However, a successful application of this proposal would require the possibility to measure concentrations of 5-FU within one hour. Conventional methods like liquid chromatography–tandem mass spectrometry (LC-MS/MS), which, although highly sensitive and robust, require complex instrumentation and longer processing times, rendering them less practical for routine clinical use and the rapid turnover that is needed in this case [39]. In contrast, immunoassay-based methods are both rapid and efficient, providing results within an hour [39]. For example, the My5-FU immunoassay, a competitive, homogeneous, two-reagent nanoparticle agglutination immunoassay [37], is a rapid testing method that takes approximately **11 min** to perform and can analyze up to **400 patient samples per hour** when used with an automated clinical chemistry analyzer. This makes it ideal for timely adjustments to treatments [40].

## 4. Materials and Methods

### 4.1. Data Search

To evaluate the contribution of the PK metrics C_ss_ and AUC to 5-FU toxicity, we screened the literature for individual patient data on the treatment schedule (dose and mode of administration) and toxicity outcomes, as well as for data on measured concentrations and/or AUC and toxicity outcomes. For the first dataset, we predicted the AUC and C_ss_ using a PBPK model for 5-FU, while for the second dataset, we calculated the related concentrations. Four relevant studies with information on both dosing as well as toxicity outcomes were identified [17,24,29,30].

### 4.2. Model-Independent Estimations of C_SS_ from Data on Dosing, Corresponding Exposure and Toxicity Outcomes

In three studies [17,29,30], dosing data as well as exposure parameters were available (AUC) (*n* = 321). Therefore, we could calculate the C_ss_ using the given infusion time and AUC values according to the formula:

C_ss_ = AUC/infusion time (1)

The AUCs were extracted using WebPlotdigitizer version 3.4 (https://automeris.io/WebPlotDigitizer/ accessed on 30 October 2024) from the figures of the respective studies [17,29,30]. This calculation might imply a small error because the C_ss_ is reached around 5 h when the drug is given by intravenous infusion. However, most of the AUC values we found were calculated using the concentration in plasma at a time point when the steady state was nearly reached.

### 4.3. Model-Dependent Estimations of C_SS_ and Auc from Data on Dosing and Corresponding Toxicity Outcomes

#### 4.3.1. Model Development and Evaluation

The PBPK structural model used is constructed of several tissue compartments that represent organs/tissues and include arterial blood and venous blood. The organs are connected with the arterial blood from which blood flows into the organs/tissues and with the venous blood to which blood flows out of the organs. The circulation system is closed via the lungs and the heart. The rate of change of the concentration is described using the following equations:

(2) $$V_{T}\frac{d}{dt}C_{T}=Q_{T}\left(C_{A}-C_{VT}\right)\;\mathrm{in}\;\text{non-metabolizing}\;\mathrm{tissues}$$

(3) $$V_{T}\frac{d}{dt}C_{T}=Q_{T}\left(C_{A}-C_{VT}\right)-RAM\;\mathrm{in}\;\mathrm{metabolizing}\;\mathrm{tissues}$$

where the following hold true:

V_T_: the volume of tissue T;

C_T_: the concentration in tissue T;

Q_T_: the blood flow through tissue T;

C_A_: the concentration in the arterial blood.

The concentration in the venous blood leaving the tissue is described by:

(4) $$C_{VT}=\frac{C_{T}}{P_{T}}$$

where P_T_ is the tissue/blood partition coefficient. 

**Figure 6:**
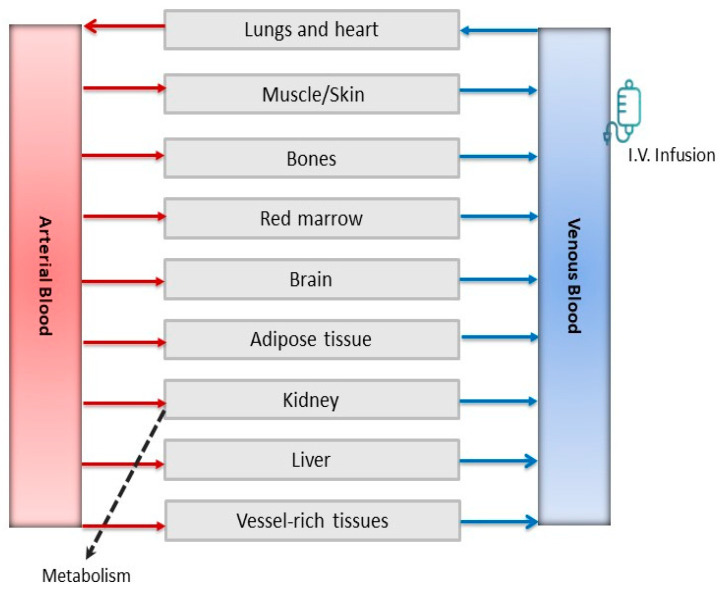
Structure of 5-FU physiologically-based pharmacokinetic model.

All calculations were conducted using R version 4.3.3 (https://www.R-project.org/ accessed on 30 October 2024) with the R package rxode2 version 2.1.2. The model code is provided in the Supplementary Material S1. The structure of the model is shown in Figure 6. The algorithm of Schmidt et al. [41] was employed to calculate the P_T_ between blood and tissues. This biologically-based algorithm is recommended in the joint EPAA–EURL ECVAM ADME workshop report and is applicable for bases, acids, neutral substances and zwitterions [42].

As 5-FU is eliminated mainly via hepatic metabolism by DHP, we decided to only include hepatic elimination in the model. According to a systematic literature search [43], three published PK studies [44,45,46] derived *K*_m_ and V_max_ in a population PK approach using a 2-compartment structural model. We used the mean values of the studies [44,45,46], with a *K*_m_ of 11.1 and a V_max_ of 1221.7 mg/h. Several polymorphisms in the dihydropyrimidine dehydrogenase gene (*DPYD*) were described; however, the polymorphism, which might reduce the activity of the enzyme to a therapeutically relevant extent, is below 1% in the European population [5]. Hence, we did not consider this group in our modeling. The physiological parameters were taken from the ICRP [47]. The in vitro and in vivo input parameters used to parameterize the models are summarized in Table 1. The appropriateness of the model and the selected parameters was confirmed by comparing the model-predicted concentration–time profile with published experimental data [27,28].

#### 4.3.2. Model-Dependent Estimations

We used the individual information on the doses, mode and duration of the administration given in the publication [24] (*n* = 138) to simulate concentration–time profiles, model the C_ss_ and derive the AUC by integration using the PBPK model. This collection of data included historical cases that experienced 5-FU overdoses, where patients were treated with high doses via short-term infusions.

This model was also employed using the data on the doses and infusion durations reported (3052 mg 5-FU over 2 h) and planned (4612 mg 5-FU over 24 h) in our patient, who was not DPD deficient, to simulate the patient’s concentration–time course with the administered erroneous schedule and therapeutic schedule as planned. The AUC and maximum concentrations were calculated in both scenarios and compared.

### 4.4. Evaluation of the Relationship Between Toxicity, C_ss_ and AUC

We evaluated the relationship in a tri-dimensional plot, with C_ss_ and AUC as the axes of the bi-dimensional plot, adding the third dimension by introducing two different colors (no toxicity, blue dots; toxicity, red dots). We also explored whether the AUC above a certain plasma concentration would be a better parameter to predict toxicity. 

### 4.5. Decision Tree

For defining whether a threshold for life-threatening toxicity is a stronger predictor for a steady-state concentration or for the AUC and at what level it can be defined, a classification tree of depth 1 was constructed using the R package rpart [48]. A classification tree provides a tree of yes/no questions (tests) on a case that result in a predicted classification for the case. Choosing depth 1 means seeking the single optimal test for predicting the true class.

**Table 1:** PBPK model parameters.

Physiological Data		
Cardiac output (Qc) (L/h) [47]	401.7	
Body weight (bw) (kg)	73	
	Blood flow through the organs (L/h) [47]	Organ volumes (L) [47]
Fat	19.5	18.2
Liver	99.5	1.8
Brain	46.8	1.45
Kidney	74.1	0.31
Muscle	65.8	0.40 × bw
Skin	20	0.0371 × bw
Vessel-rich tissue (excluding red marrow)	44.8	2.6
Skeleton	7.8	6.9
Red marrow	11.7	1.2
**Substance-Specific Data**		
Log K_o/w_ [49]	−0.89	
pKa [49]	8.02	
Protein binding (%) [49]	8–12	
Partition coefficient ^1^		
Fat/blood	0.20	
Liver/blood	0.92	
Brain/blood	1.35	
Kidney/blood	0.98	
Muscle	2.19	
Skin	10.04	
Vessel-rich tissue (excluding red marrow)	0.98	
Skeleton	0.63	
Red marrow	0.92	
Metabolic constants ^2^		
V_max_ (mg/h)	1221.7	
K_m_ (mg/L)	11.7	

^1^ calculated according to the algorithm of Schmidt [42]. ^2^ mean of values reported by [44,45,46].

## 5. Conclusions

The 5-FU steady-state plasma concentration is a better toxicity predictor than the AUC. In patients for whom higher doses than recommended in the existing protocols are clinically considered relevant, monitoring the concentration at one hour is recommended. At this time point, concentrations above 1.5 mg/L are likely predictive of non-lethal toxicity, while those above 3 mg/L are predictors of lethal toxicity. Monitoring the concentration at one hour after the infusion is started has the advantage that a dose correction could be made early before toxicity is observed.

## Data Availability

The original data, cited in the article, are publicly available as figures. The data are obtained from original literature data using WebPlotdigitizer version 3.4 and can be made available on request from the corresponding author.

## Supplementary Materials

The following supporting information can be downloaded at: https://www.mdpi.com/article/10.3390/ph18050653/s1, Supplementary Material S1: R script for 5-FU PBPK model code.

## Abbreviations

The following abbreviations are used in this manuscript:

5-FU	5-fluorouracil
AUC	Area under the plasma concentration–time curve
PBPK	Physiologically-based pharmacokinetic modeling
TS	Thymidylate synthase
DPD	Dihydropyrimidine dehydrogenase
TDM	Therapeutic drug monitoring
IATDMCT	International Association of Therapeutic Drug Monitoring and Clinical Toxicology
PK	Pharmacokinetic
C*_ss_*	Steady-state concentration
LC-MS/MS	Liquid chromatography–tandem mass spectrometry
