# Supplementary material for: 5-Fluorouracil Toxicity: Revisiting the Relevance of Pharmacokinetic Parameters
Source: Pharmaceuticals (Basel). 2025 Apr 29;18(5):653. doi: 10.3390/ph18050653 (PMC12114802; doi:10.3390/ph18050653)
Supplement: Supplementary file 1 [file pharmaceuticals-18-00653-s001.zip › pharmaceuticals-3529141-supplementary.pdf]

5-FU PBPK model code

```
library(rxode2)
MW = 130.08    # molecular weight of 5-FU (g/mol)
params <- c(
  ####################################
  ###  Physiological parameters  ###
  ####################################

  # Tissue volumes
  VF  = 18.200,        # Volume fat tissue (l)
  VL  = 1.800,         # Volume liver (l)
  VBr = 1.450,         # Volume brain (l)
  VK  = 0.310,         # Volume kidney (l)
  VMS = 32.300,        # Volume muscle/skin (l)
  VVR = 3.768 - 1.170,  # Volume vessel rich tissue (l), minus red bone marrow
  VRM = 1.170,         # volume red marrow (l)
  VSkel = 9.330,       # Volume skeleton (l)

  # Blood flows
  QF  = 19.5,          # Blood flow to fat (l/h)
  QL  = 99.5,          # Blood flow to liver (l/h)
  QBr = 46.8,          # Blood flow to brain (l/h)
  QK  = 74.1,          # Blood flow to kidney (l/h)
  QMS = 85.8,          # Blood flow to muscle/skin (l/h)
  QVR = 56.5 - 11.7,    # Blood flow to vessel rich tissue (l/h), minus red bone marrow
  QRM = 11.7,          # Blood flow to red marrow (l/h)
  QSkel = 7.8,         # Blood flow to skeleton (l/h)

  # hypothetic infusion 1 l/h
  Qinf = 1,
```

```
#######################################
###  Chemical-specific parameters  ###
#######################################

# Partition coefficients for 5-FU
# according to Schmitt

PL   = 0.92,       # Liver/blood partition coefficient
PF   = 0.2,        # Fat/blood partition coefficient
PBr  = 1.35,       # Brain/blood part. coeff.  (rapidly perfused)
PK   = 0.98,       # Kidney (rapidly perfused)
PMS  = 2,          # Muscle/skin (slowly perfused) (Schmitt: muscle 2.19, skin 1.04)
PVR  = 0.92,       # vessel rich (rapidly perfused) (wie Leber)
PRM  = 0.9,        # red marrow (from Schmitt [3] red blood cells)
PSkel= 0.63,       # Skeleton (slowly perfused)

# Data for metabolism, we use the mean of three sources
# VMAX = 1390, KM =  5.57    [4]
# VMAX = 1512, KM =  4.55    [5]
# VMAX =  763, KM = 25       [6]
#
# [4] Terret, C., Erdociain, E., Guimbaud, R. et al. Dose and time
#     dependencies of 5-fluorouracil pharmacokinetics, Clin Pharmacol Ther,
#     68: 270-9 (2000) https://doi.org/10.1067/mcp.2000.109352
# [5] van Kuilenburg, A. B., Häusler, P., Schalhorn, A. et al. Evaluation
#     of 5-fluorouracil pharmacokinetics in cancer patients with a c.1905+1G>A
#     mutation in DPYD by means of a Bayesian limited sampling strategy,
#     Clin Pharmacokinet, 51: 163-74 (2012) https://doi.org/10.1007/bf03257473
# [6] Woloch, C., Di Paolo, A., Marouani, H. et al. Population
#     pharmacokinetic analysis of 5-FU and 5-FDHU in colorectal cancer
#     patients: search for biomarkers associated with gastro-intestinal
#     toxicity, Curr Top Med Chem, 12: 1713-9 (2012)
```

```
   #    https://doi.org/10.2174/156802612803531414

   VMAX = mean(c(1390, 1512, 763)),       # Maximum velocity of metabolism (mg/h)
   KM = mean(c(5.57, 4.55, 25))           # Michaelis-Menten  (mg/l)
)

# calculate Cardiac output (l/h)
params <- c(params,
         QC = with(as.list(params), QF + QBr + QK + QMS + QVR + QRM + QSkel + QL))


############################
###  Equations and ODEs  ###
############################

ode <- "
   # 5-FU in fat compartment
   CF = AF/VF                      # (mg/l)
   CVF = CF/PF                     # Venous blood (mg/l)

   # 5-FU in brain compartment
   CBr = ABr/VBr                   # (mg/l)
   CVBr = CBr/PBr                  # Venous blood (mg/l)

   # 5-FU in kidney compartment
   CK = AK/VK                      # (mg/l)
   CVK = CK/PK                     # Venous blood (mg/l)

   # 5-FU in 'Muscle/Skin' compartment
   CMS = AMS/VMS                   # (mg/l)
   CVMS = CMS/PMS                  # Venous blood (mg/l)
```

```
# 5-FU in vessel rich compartment & red marrow
CVR = AVR/VVR                    # (mg/l)
CVVR = CVR/PVR                   # Venous blood (mg/l)
CRM = ARM/VRM                    # (mg/l)
CVRM = CRM/PRM                   # Venous blood (mg/l)

# 5-FU in skeleton compartment w/o bone marrow
CSkel = ASkel/VSkel              # (mg/l)
CVSkel = CSkel/PSkel             # Venous blood (mg/l)

# 5-FU in liver compartment
CL = AL/VL                       # (mg/l)
CVL = CL/PL                      # Venous blood (mg/l)

# 5-FU in blood
d/dt(iv.dose) = 0
CV = (QF*CVF + QBr*CVBr + QK*CVK + QMS*CVMS + QVR*CVVR + QRM*CVRM +
      QSkel*CVSkel + QL*CVL)/QC            # Mixed venous (mg/l)
CA = (QC*CV + Qinf*iv.dose)/(QC+Qinf)      # Arterial (mg/l)
                        # iv.dose in 1L hypothetical Infusion

# 5-FU metabolism
# Michaelis-Menten
RAM =  VMAX*CL/(KM+CL)           # (mg/h)

# Mass balance for 5-FU
MASS = AF + ABr + AK + AMS + AVR + ARM + ASkel + AL + AM
                        # In tissues + metabolized (mg)

d/dt(AF) = QF*(CA-CVF)           # (mg/h)
d/dt(ABr) = QBr*(CA-CVBr)        # (mg/h)
d/dt(AK) = QK*(CA-CVK)           # (mg/h)
```

```
  d/dt(AMS) = QMS*(CA-CVMS)          # (mg/h)
  d/dt(AVR) = QVR*(CA-CVVR)          # (mg/h)
  d/dt(ARM) = QRM*(CA-CVRM)          # (mg/h)
  d/dt(ASkel) = QSkel*(CA-CVSkel)    # (mg/h)
  d/dt(AL) = QL*(CA-CVL) - RAM       # (mg/h)
  d/dt(AM) = RAM                     # (mg/h)
  d/dt(AUC) = CA                     # (mg/l)
  d/dt(AUCRM) = CRM                  # (mg/l)
"

iv.model <- rxode2(model = ode)

inits <- c(
  ADC   = 0,            # (mg)
  AF    = 0,            # (mg)
  ABr   = 0,            # (mg)
  AK    = 0,            # (mg)
  AMS   = 0,            # (mg)
  AVR   = 0,            # (mg)
  ARM   = 0,            # (mg)
  ASkel = 0,            # (mg)
  AL    = 0,            # (mg)
  AM    = 0,            # (mg)
  AUC   = 0,            # (mg/l * h)
  AUCRM = 0,            # (mg/l * h)
  iv.dose = 1e-6        # (mg) will be adapted to scenario
)

################################
###  Simulation of patient  ###
################################
```

```
## Simulation for actual dosing
iv.dose <- 3052   # [mg]
iv.duration <- 2  # [h]
inits["iv.dose"] = iv.dose/iv.duration

qd <- eventTable(amount.units = "ug", time.units = "hours")
qd$add.sampling(seq(from = 0, to = 30, by = 0.1))
qd$add.dosing(dose = -iv.dose/iv.duration,
        dosing.to = "iv.dose",
        start.time = iv.duration)

actual_dosing <- rxSolve(iv.model, params = params, events = qd,
              inits = inits, method = "lsoda")
# plot results: 'plot(actual_dosing)' or 'plot(actual_dosing, "CA")'

## Simulation for intended dosing
iv.dose <- 4612   # [mg]
iv.duration <- 24  # [h]
inits["iv.dose"] = iv.dose/iv.duration

qd <- eventTable(amount.units = "ug", time.units = "hours")
qd$add.sampling(seq(from = 0, to = 30, by = 0.1))
qd$add.dosing(dose = -iv.dose/iv.duration,
        dosing.to = "iv.dose",
        start.time = iv.duration)

intended_dosing <- rxSolve(iv.model, params = params, events = qd,
               inits = inits, method = "lsoda")

# Extract numerical values after 3 hours (as an example)
idx3 <- which(actual_dosing$time==units::set_units(3, "hours"))
```

```
idx24 <- which(actual_dosing$time==units::set_units(24, "hours"))
actual_dosing[idx3, "CRM"]
actual_dosing[idx3, "CA"]
intended_dosing[idx24, "AUC"]

# generate figure 3 for publication
pdf("Fig_3.pdf", width = 6, height = 4)
patientin <- data.frame(time = actual_dosing$time,
                    given = actual_dosing$CA,
                    planned = intended_dosing$CA)
plot(given ~ time, data = patientin, col = "red", type = "l", lwd = 2, ylab = "mg/L")
lines(planned ~ time, data = patientin, col = "blue", lwd = 2)
legend(25, 25, xjust = 1, yjust = 1,
    legend=c(sprintf("AUC given %.1f mg/Lxh", max(actual_dosing$AUC)),
        sprintf("AUC planned %.1f mg/Lxh", max(intended_dosing$AUC)),
        sprintf("Cmax given %.1f mg/L", max(actual_dosing$CA)),
        sprintf("Cmax planned %.1f mg/L", max(intended_dosing$CA))))
# Note this is the total AUC, not the 24 hour AUC.
# (Actually, it is the 30 hour AUC, but after that time CA is essentially 0.)
dev.off()
```
